# Xanthohumol Protects the Rat Myocardium against Ischemia/Reperfusion Injury-Induced Ferroptosis

**DOI:** 10.1155/2022/9523491

**Published:** 2022-01-17

**Authors:** Jian-Hong Lin, Kun-Ta Yang, Wen-Sen Lee, Pei-Ching Ting, Yu-Po Luo, Ding-Jyun Lin, Yi-Shun Wang, Jui-Chih Chang

**Affiliations:** ^1^Division of Experimental Surgery, Department of Surgery, Hualien Tzu Chi Hospital, Buddhist Tzu Chi Medical Foundation, No. 707, Sec. 3, Zhongyang Rd., Hualien, Taiwan; ^2^Department of Physiology, School of Medicine, Tzu Chi University, No. 701, Sec. 3, Zhongyang Rd., Hualien, Taiwan; ^3^Graduate Institute of Medical Sciences, College of Medicine, Taipei Medical University, No. 250, Wuxing St., Taipei, Taiwan; ^4^Department of Surgery, Hualien Tzu Chi Hospital, Buddhist Tzu Chi Medical Foundation, No. 707, Sec. 3, Zhongyang Rd., Hualien, Taiwan; ^5^School of Medicine, Tzu Chi University, No. 701, Sec. 3, Zhongyang Rd., Hualien, Taiwan; ^6^Department of Life Sciences, Tzu Chi University, No. 701, Sec. 3, Zhongyang Rd., Hualien, Taiwan; ^7^Department of Surgery, School of Medicine, Tzu Chi University, No. 701, Sec. 3, Zhongyang Rd., Hualien, Taiwan

## Abstract

Ferroptosis is an iron-dependent form of cell death caused by the inactivation of glutathione peroxidase 4 (GPX4) and accumulation of lipid peroxides. Ferroptosis has been found to participate in the ischemia-reperfusion (I/R) injury, leading to heart dysfunction and myocardial cell death. Xanthohumol (XN), a prenylated flavonoid isolated from *Humulus lupulus*, has multiple pharmacological activities, such as anti-inflammatory and antioxidant. This study is aimed at investigating whether XN could attenuate the I/R-induced ferroptosis in cardiomyocytes and the underlying mechanisms. Cardiomyocytes were treated with Fe-SP and RSL3, and the rat hearts were treated with I/R. The results from the present study show that XN was able to protect cardiomyocytes against Fe-SP- and RSL3-induced ferroptotic cell death by decreasing the production of lipid peroxidation and ROS, chelating iron, reducing the NRF2 protein level, and modulating the protein levels of GPX4. Moreover, XN significantly decreased the mRNA levels of ferroptosis markers, *Ptgs2* and *Acsl4*, and the protein levels of ACSL4 and NRF2 and modulated the protein levels of GPX4 in I/R-treated hearts. The findings from the present study suggest that XN might have the therapeutic potential for the I/R-induced ferroptosis injury.

## 1. Introduction

According to the *World Health Organization*, ischemic heart disease is the first leading cause of death worldwide [1]. Currently, reperfusion is the definitive treatment for acute ischemic heart disease [2]. However, there is no effective therapy to prevent heart damage caused by ischemia-reperfusion (I/R) injury (IRI). IRI may cause massive cardiomyocyte death, which is a major cause of cardiac dysfunction, leading to cardiac arrhythmias, heart failure, and death [3]. Oxidative stress has been demonstrated to consider a key initiator of IRI in clinical [4]. In addition, the deregulation of iron homeostasis has an important contribution to increasing intracellular free iron in cardiac cells [5]. The iron ion can enhance the generation of reactive oxygen species (ROS) by Fenton reaction, subsequently increasing cell damage through the attack of lipids to induce ferroptosis, which differs from apoptosis, necrosis, or autophagy [6].

Ferroptosis is an iron-dependent form of cell death caused by inactivation of glutathione peroxidase 4 (GPX4) and accumulation of lipid peroxides [7]. GPX4 not only catalyzes the peroxidation of hydrogen peroxide (H_2_O_2_) but also detoxifies the lipid peroxides by reducing glutathione [8]. Jang et al. reported that ferroptotic stimuli reduce mitochondrial bioenergetics, glutathione depletion, and GPX4 inactivation, leading to ferroptosis in cardiomyocytes [9]. Park et al. indicated that downregulation of GPX4 during myocardial infarction contributes to ferroptotic cell death in cardiomyocytes upon metabolic stress [10]. Feng et al. reported that the liproxstatin-1 (Lip-1), a ferroptosis inhibitor, protects the myocardium against IRI by protecting mitochondrial structural integrity and function and restoring the GPX4 level to reduce heart damage [11].

Under physiological conditions, almost all iron is tightly bound to iron-protein, such as transferrin and ferritin, which control iron reactivity. During ischemia, however, a large amount of iron is released from iron-protein and then participates in the generation of free radicals [12]. Previous studies have shown that iron chelators can protect the heart from IRI. Tang et al. showed that deferoxamine, an iron chelator, significantly attenuated myocardial injury in the I/R-treated rat hearts [13]. Fang et al. demonstrated that inhibition of ferroptosis by dexrazoxane, an iron chelator, or ferrostatin-1 (Fer-1), a ferroptosis inhibitor, reduces the myocardial infarct size through maintaining mitochondrial function [14].

Safe and effective natural products play a vital role in drug discovery, especially for cardiovascular diseases [15–18]. Flavonoids are naturally presented in a variety of fruit, vegetables, and plant-based food products. Flavonoids can not only inhibit lipid peroxidation and chelate redox-active metals but also improve cardiac function and attenuate myocardial infarction [19–21]. Recently, it has been reported that flavonoids can suppress erastin-induced ferroptosis in human pancreatic ductal adenocarcinoma cells [22]. In addition, epidemiological studies have shown a positive correlation between the diet of flavonoid-rich foods and cardiovascular health [23]. Xanthohumol (XN), a prenylated flavonoid isolated from *Humulus lupulus*, has multiple bioactivities, such as anti-inflammatory, antioxidant, and antilipid peroxidation, as well as antiatherosclerosis, antiadipogenesis, and anticancer [24]. However, the mechanisms underlying XN-induced protection against ferroptosis remain unclear. The object of the present study was to investigate whether XN could attenuate the I/R-induced ferroptosis in cardiomyocytes and the underlying mechanisms of its action.

## 2. Materials and Methods

### 2.1. Chemicals

XN was purchased from Selleckchem.com (Houston, TX). Dimethyl sulfoxide (DMSO) and 1, 10-phenanthroline (phen) were purchased from Sigma-Aldrich (St. Louis, MO). Lip-1, Fer-1, (1S,3R)-RSL3, and Chlorido [N, N′-disalicylidene-1,2-phenylenediamine]iron (III) (Fe-SP) were purchased from Cayman Chemical Company (Ann Arbor, MI).

### 2.2. H9c2 Cardiomyoblasts Culture

H9c2 cardiomyoblasts obtained from ATCC were cultured in Dulbecco's Modified Eagle Medium-high glucose (Gibco, Grand Island, NY) supplemented with 10% fetal bovine serum (Gibco) and 1% penicillin/streptomycin (Gibco). H9c2 cardiomyoblasts were seeded in 0.1% gelatin-coated plate and incubated at 37°C in a fully humidified atmosphere with 5% CO_2_, and the medium was changed every 2 days. After growing to 80% confluence, the cells were rinsed with phosphate-buffered saline (PBS) solution, detached using 0.25% trypsin-EDTA (Gibco), and then, neutralized by adding fresh medium. The cell suspension was centrifuged at 1,200 rpm for 5 min. The cell pellet was resuspended with the medium, and dispensed into a new 15 cm petri dish.

### 2.3. Preparation and Culture of Ventricular Myocytes

All laboratory animal protocol was approved by the Institution of Animal Care and Use Committee of the Hualien Tzu Chi Hospital (IACUC Approval No. 108-10). Two-day-old neonatal Sprague-Dawley rats (both sexes) were used as cardiomyocyte donors and sacrificed by decapitation according to a previous report [25]. Briefly, the isolated hearts were washed using Hank's buffer (Gibco). The tissues were minced and digested with 0.045% pancreatin (Sigma) and 0.01% collagenase II (Gibco) in Hank's buffer, and the enzyme was then inactivated with culture medium (F-12 medium supplemented with 10% fetal bovine serum, 10% horse serum, and 1% penicillin/streptomycin; Gibco). Cells were plated onto 10 cm petri dish and incubated for 1 h at 37°C in a fully humidified atmosphere with 5% CO_2_ incubator to reduce fibroblast contamination. The ventricular myocytes were collected and seeded on 0.1% collagen-coated 6 cm petri dish in culture medium with 10 *μ*M cytosine arabinoside, and the medium was daily changed.

### 2.4. Langendorff Heart Perfusion System

Rats were anesthetized with urethane (1.5 g/kg, i.p.), then hearts were excised and arrested in Krebs Henseleit (KH) buffer as previously described [26]. Following 30 min equilibration, ischemia was induced by halting perfusion for 45 min. Reperfusion was followed with KH buffer and XN (5 or 10 *μ*M) together for 60 min. Control hearts were not subjected to I/R.

### 2.5. Cell Viability Assay

H9c2 cardiomyoblasts and cardiomyocytes were seeded in 24-well plate. After 16 h, the medium was removed, and 3-(4,5-dimethylthiazol-2-yl)-2,5-diphenyl tetrazolium bromide (MTT) (Sigma-Aldrich) solution (5 mg/mL in PBS) was added to each 24-well plate and incubated for 2 h at 37°C. Then, formazan salts were solubilized with DMSO and absorbance at 570 nm was measured using an ELISA plate reader (Multiskan EX; Thermo, Waltham, MA). The absorbance of each well was measured at 570 nm using an ELISA plate reader (Multiskan EX; Thermo, Waltham, MA).

### 2.6. Cell Death Analysis

First, the treated cells were trypsinized, collected and stained with Annexin V-FITC Apoptosis Detection Kit (BioVision, CA) in binding buffer, and detected by Gallios™ Flow Cytometer (Beckman Coulter, Brea, CA). The excitation/emission was detected at 488/525 nm wavelength, and PI emission was detected at 575 nm wavelength. Second, Hoechst 33342 (Sigma-Aldrich) and propidium iodide (PI) (Sigma-Aldrich) double staining were used to validate any changes in apoptosis, necrosis, and cell populations. Apoptosis was characterized by chromatin condensation, whereas necrosis was stained with PI. The cells were incubated with N-2-hydroxy-ethylpiperazine-N′-2-ethanesulphonic acid- (HEPES-) buffered Tyrode solution (NT) containing Hoechst 33342 and PI for 30 min at 37°C. After staining, the cells were washed three times with NT and then fixed in 10% formaldehyde for 1 h at 37°C. Images were captured at 40x magnifications using a confocal microscope (Nikon C2 Si^+^; Melville, NY) and analyzed using NIS-Elements imaging software.

### 2.7. Reactive Oxygen Species (ROS) Analysis

H9c2 cardiomyoblasts were incubated with N-2-hydroxy-ethylpiperazine-N′-2-ethanesulphonic acid- (HEPES-) buffered Tyrode solution (NT) containing 2.5 *μ*M CM-H_2_DCFDA (Molecular Probes; Eugene, OR) for 30 min at 37°C. Intracellular ROS analyses were carried out using Gallios™ Flow Cytometer. The excitation/emission was detected at 488/525 nm wavelength. For confocal images, cells were incubated with NT containing 2.5 *μ*M CM-H_2_DCFDA and Hoechst 33342 for 30 min at 37°C. Images were captured at 40x magnifications using a confocal microscope and analyzed using NIS-Elements imaging software.

### 2.8. Iron Content Analysis

The Phen Green SK (PGSK) diacetate (Cayman Chemical Company) was used to monitor the content of intracellular Fe^2+^ by Gallios™ Flow Cytometer. The fluorescence of the dye PGSK is quenched upon interaction with Fe^2+^. H9c2 cardiomyoblasts were incubated with PBS containing 2 *μ*M PGSK for 30 min at 37°C. The excitation/emission was detected at 488/525 nm wavelength.

### 2.9. Thiobarbituric Acid Reactive Substances (TBARS) Assay

H9c2 cardiomyoblasts were seeded in 0.1% gelatin-coated 10 cm petri dish. Lipid peroxidation was evaluated by TBARS assay kit (Cayman Chemical Company) following manufacturer's instructions. Briefly, cells were washed with PBS several times, collected, and sonicated. After adding SDS solution to vial, the mixture was swirled, added with color reagent, boiled for 1 h, incubated on ice for 10 min, and then, centrifuged at 1,600 × g for 10 min at 4°C. The plate was read fluorescence at excitation 530/emission 555 nm wavelength by multimode microplate reader (Varioskan LUX; Thermo).

### 2.10. Lipid Peroxidation Analysis

H9c2 cardiomyoblasts were seeded in 24 mm coverslips coated with 0.1% collagen and incubated with NT containing 2 *μ*M C11 BODIPY581/591 (Cayman Chemical Company) and Hoechst 33342 for 30 min at 37°C. Images were captured at 40x magnifications using a confocal microscope and analyzed using NIS-Elements imaging software. For cardiomyocytes, the cells were seeded in 24 mm 0.1% collagen-coated coverslips. After 24 h, the cells were incubated with NT containing 2 *μ*M C11 BODIPY581/591 for 1 h at room temperature. Images were captured at 40x magnifications using a confocal microscope and analyzed using NIS-Elements imaging software.

### 2.11. Western Blot Analysis

Cells were washed twice with PBS and lysed in RIPA lysis buffer (Millipore) containing protease inhibitor (Calbiochem) and phosphatase inhibitor (Calbiochem). The lysates were clarified by centrifugation at 12,000 rpm for 15 min at 4°C, and the protein concentration was determined using a BSA protein assay kit (Bio-Red; Hercules, CA). Western blot analysis was performed according to a previous report [27]. Briefly, the sample containing 30 *μ*g of protein was loaded into a 10% SDS-polyacrylamide gel and then transferred to PVDF membrane (Millipore). The membrane was treated with 5% BSA to block the nonspecific binding of IgG and then incubated with the primary antibodies overnight at 4°C. Anti-GPX4 (Cat No. GTX54095), anti-FTH (Cat No. GTX101733), and anti-NRF2 (Cat No. GTX103322) purchased from GeneTex (Irvine, CA) were in 1,000 dilution. Anti-ACSL4 (Cat No. ab155282) purchased from Abcam (Cambridge) was in 1,000 dilution. Anti-*β*-actin (Cat No. MAB1501) purchased from Millipore was in 10,000 dilution. Anti-GAPDH (Cat No. GTX100118) purchased from GeneTex was in 10,000 dilution. After washing, the membrane was incubated with the secondary antibodies (peroxidase-conjugated goat anti-rabbit IgG or anti-mouse IgG) at a 1 : 5,000 dilution. The detected proteins were visualized with an ECL detection kit (Cat No. GERPN2232, GE Healthcare; Uppsala, Sweden) and UVP ChemStudio Plus touch (Analytik Jena; Jena, Germany). The resulting bands were analyzed using Image-Pro Plus 4.5 software (Media Cybernetics, Silver Spring, USA) and normalized using the level of *β*-actin or GAPDH.

### 2.12. Quantitative Real-Time PCR

Total RNAs were extracted from H9c2 cardiomyoblasts using the Trizol reagent (Ambion; Carlsbad, CA) according to manufacturer's instructions. The cDNA was synthesized with Verso™ cDNA Kit (Thermo) using 3 *μ*g of total RNAs. Quantitative real-time PCR was performed with the LightCycler 480 System (Roche; Basel, Switzerland) using Fast SYBR Green Master Mix (Thermo). The following PCR primers were used: *Ptgs2* forward: 5′-ATG TTC GCA TTC TTT GCC CAG-3′; *Ptgs2* reverse: 5′-TAC ACC TCT CCA CCG ATG AC-3′; *Acsl4* forward: 5′-TCC AAG CCA GAA AAC TCA AGC-3′; *Acsl4* reverse: 5′-GGT GTA CAT GAC AAT GGC CAT-3′; *Gapdh* forward: 5′-ATG TTC CAG TAT GAC TCC ACT CAC G-3′; *Gapdh* reverse: 5′-GAA GAC ACC AGT AGA CTC CAC GAC A-3′. All gene expression was analyzed using the comparative *C*_*t*_ method (2^−ΔΔCt^), where ΔΔ*C*_*t*_ = Δ*C*_*t*_ (sample) − Δ*C*_*t*_ (reference) relative to *Gapdh* levels.

### 2.13. Statistics

Experimental data were presented as means ± standard error of the mean (SEM). Comparisons were subjected to one-way ANOVA followed by Fisher's least significant difference test, and *p* values < 0.05 were considered significant.

## 3. Results

### 3.1. Effects of XN on Ferroptotic Cell Death

**Figure 1:**
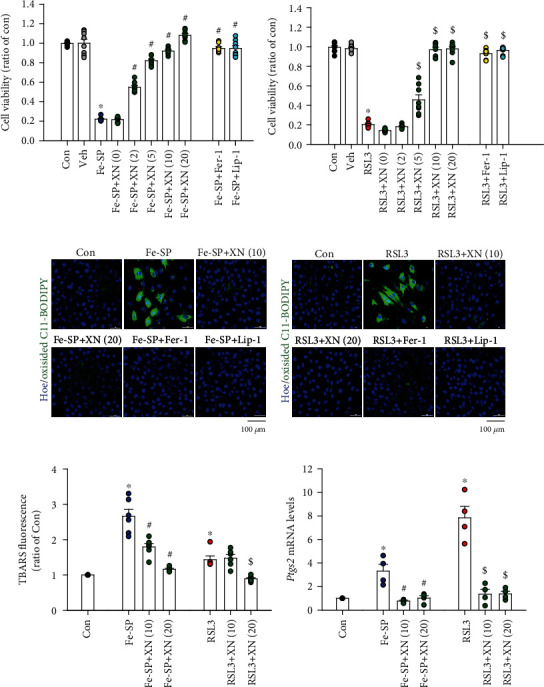
XN protects H9c2 cardiomyoblasts against ferroptotic cell death. Treatment with (a) Fe-SP (0.5 *μ*M) or (b) RSL3 (0.1 *μ*M) for 16 h decreased the viability of H9c2 cardiomyoblasts detected using the MTT assay, and this effect was inhibited by cotreatment with XN (5-20 *μ*M) or a ferroptosis inhibitor (ferrostatin-1, Fer-1, 1 *μ*M or liproxstatin-1, Lip-1, 2 *μ*M) (*n* = 8). Confocal images of H9c2 cardiomyoblasts treated with (c) Fe-SP (0.5 *μ*M) or (d) RSL3 (0.1 *μ*M) for 16 h were measured using Hoechst 33342 and lipid ROS probe C11 BODIPY 581/591. A significant increase of the lipid peroxidation was observed (green), and this effect was abolished by XN (10 or 20 *μ*M), Fer-1 (1 *μ*M), or Lip-1 (2 *μ*M). Fe-SP (0.5 *μ*M) or RSL3 (0.1 *μ*M) reduced H9c2 cardiomyoblasts number, but did not induce chromatin condensation. Scale bars = 100 *μ*m. (e) Treatment with Fe-SP or RSL3 for 16 h enhanced the malondialdehyde content in H9c2 cardiomyoblasts detected using the TBARS assay, and this effect was inhibited by cotreatment with XN (10 or 20 *μ*M) (*n* = 6). (f) XN (10 or 20 *μ*M) significantly decreased the levels of *Ptgs2* mRNA in H9c2 cardiomyoblasts-treated with Fe-SP or RSL3 for 16 h, was measured using real-time PCR (*n* = 4). All data represent mean ± SEM. ^∗^*p* < 0.05, versus the control group; ^#^*p* < 0.05, versus the Fe-SP; ^$^*p* < 0.05, versus the RSL3. Con: control; Veh: vehicle; Hoe: Hoechst 33342.

Fe-SP (0.5 *μ*M) and RSL3 (0.1 *μ*M) have been reported to induce ferroptosis through generating the lipid-based ROS and suppressing the GPX4 activity, respectively [28, 29]. Although H9c2 cardiomyoblasts were not able to beat, they still showed many similarities to primary cardiomyocytes, including membrane morphology, g-signaling protein expression, and electrophysiological properties [30]. In the present study, we used the H9c2 rat cardiomyoblast cell line, which has the advantage of being an animal-free alternative, to assess the cytoprotection effect of XN on the Fe-SP-induced H9c2 cardiomyoblasts ferroptosis using the MTT assay. As shown in Figure 1, treatment with XN (2-20 *μ*M) for 16 h significantly protected H9c2 cardiomyoblasts against the Fe-SP-induced ferroptotic cell death in a concentration-dependent manner (Figure 1(a), left panel). We also used RSL3, another ferroptosis inducer, to examine the cytoprotection effect of XN on H9c2 cardiomyoblasts. Treatment with XN (5-20 *μ*M) for 16 h also significantly protected H9c2 cardiomyoblasts against the RSL3-induced ferroptotic cell death in a concentration-dependent manner (Figure 1(b), left panel). DMSO (1 : 1,000), the vehicle used to dissolve Fe-SP and RSL3, did not significantly affect the viability of H9c2 cardiomyoblasts. We also used the ferroptosis inhibitors, Fer-1 and Lip-1, to confirm that XN protects H9c2 cardiomyoblasts against cell death through inducing ferropotosis. Our results show that both Fer-1 (1 *μ*M) and Lip-1 (2 *μ*M) significantly inhibited the Fe-SP- and RSL3-induced ferroptotic cell death, respectively (Figures 1(a) and 1(b), right panel). Ferroptosis is an iron-dependent form of cell death associated with increased lipid peroxidation. We also evaluated the effect of the XN on the ferroptotic cell death and lipid peroxidation by confocal microscopy using Hoechst 33342 and lipid ROS probe C11 BODIPY 581/591. As demonstrated in (Figures 1(c) and 1(d)), treatment with XN (10 or 20 *μ*M), Fer-1, or Lip-1 can abolish the Fe-SP- or RSL3-induced decreases of the H9c2 cardiomyoblasts number and increases of lipid peroxidation. In addition, treatment with Fe-SP or RSL3 did not induce chromatin condensation in H9c2 cardiomyoblasts. Further, we used the TBARs assay kit to detect malondialdehyde (MDA), one of the most prevalent byproducts of lipid peroxidation. The results showed that XN (10 or 20 *μ*M) can significantly inhibit the Fe-SP-induced increases in MDA concentration in H9c2 cardiomyoblasts (Figure 1(e), left panel); likewise, the XN (20 *μ*M) also can inhibit the RSL3-induced MDA enhancement (Figure 1(e), right panel). In addition, treatment with XN (10 or 20 *μ*M) significantly downregulated the mRNA levels of prostaglandin-endoperoxide synthase 2 (*Ptgs2*), a molecular marker of ferroptosis, in the Fe-SP- and RSL3-induced ferroptotic cell death in H9c2 cardiomyoblasts (Figure 1(f)). These results showed that XN ameliorated the ferroptosis-induced lipid peroxidation and protected H9c2 cardiomyoblasts against ferroptotic cell death.

### 3.2. Effects of XN on ROS Production and Iron Content

**Figure 2:**
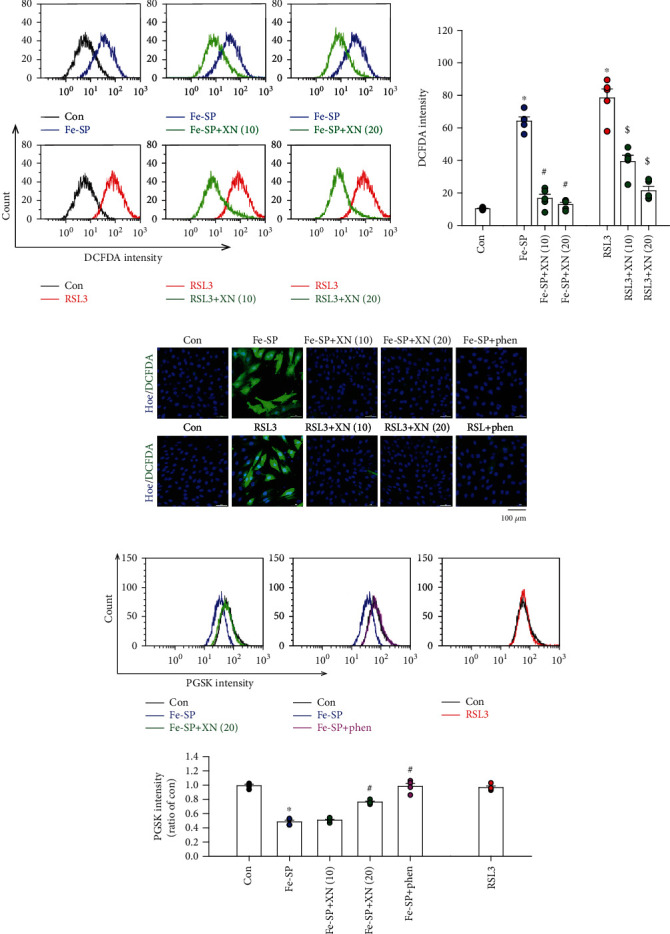
XN reduces ROS and iron accumulation in H9c2 cardiomyoblasts. (a) Treatment with Fe-SP (0.5 *μ*M) or RSL3 (0.1 *μ*M) for 16 h increased the intracellular ROS, and this effect was abolished by cotreatment with XN (10 or 20 *μ*M). Left panel shows the DCFDA intensity, a fluorescence indicator of intracellular ROS. Right panel shows a graph of quantitation of these data (n =5). (b) The ROS image was detected by confocal microscopy using CM-H_2_DCFDA. XN (10 or 20 *μ*M) or a ferrous iron chelator, phen (50 *μ*M) reduced the intracellular ROS in the Fe-SP- or RSL3-treated H9c2 cardiomyoblasts. Hoechst 33342 was used to identify the cell nuclei. Scale bars = 100 *μ*m. (c) Fe-SP, but not RSL3, increased the intracellular iron, and this effect was abolished by cotreatment with XN (20 *μ*M) or phen (50 *μ*M). Upper panel shows the PGSK intensity, a fluorescence indicator of intracellular iron. Bottom panel shows a graph of quantitation of these data (*n* = 4). All data represent mean ± SEM. ^∗^*p* < 0.05, versus the control group; ^#^*p* < 0.05, versus the Fe-SP; ^$^*p* < 0.05, versus the RSL3. Con: control; phen: 1, 10-phenanthroline; Hoe: Hoechst 33342.

Lipid peroxidation has been described as a process under which oxidants such as ROS, attack lipids [31]. In the present study, we examined the ROS production in the Fe-SP- and RSL3-treated H9c2 cardiomyoblasts. The specific ROS (H_2_O_2_ and hydroxyl radical (·OH)) measuring dye (CM-H_2_DCFDA) was used to measure the intracellular ROS by flow cytometry or confocal microscopy. As demonstrated in (Figures 2(a) and 2(b)), ROS generation was increased in H9c2 cardiomyoblasts treated with Fe-SP (0.5 *μ*M) or RSL3 (0.1 *μ*M). Cotreatment with XN (10 or 20 *μ*M) and Fe-SP or RSL3 significantly decreased the ROS generation as compared with the cells treated with Fe-SP or RSL3 alone. We also observed that phen, a ferrous ion chelator, significantly decreased the ROS generation in H9c2 cardiomyoblasts treated with Fe-SP or RSL3 (Figure 2(b)). ·OH has particularly strong oxidizability with a one-electron reduction potential to attack DNA, lipid, and proteins. In cells, ·OH can be generated by the ferrous iron-catalyzed decomposition of H_2_O_2_ via the Fenton reaction [31]. However, the role of XN in ferroptosis remains unexplored. We used flow cytometry and PGSK to detect intracellular iron concentrations. The PGSK fluorescence was quenched upon interaction with iron. As shown in Figure 2(c), treatment with Fe-SP, but not RSL3, decreased the PGSK fluorescence intensity. This result suggests the occurrence of intracellular iron accumulation. However, after the cotreatment with Fe-SP and XN (20 *μ*M), the PGSK fluorescence intensity remained the same, suggesting the XN inhibited Fe-SP-induced intracellular iron accumulation. To confirm the Fe-SP-induced intracellular iron accumulation, phen was applied to inhibit the occurrence of intracellular iron accumulation in the Fe-SP-treated H9c2 cardiomyoblasts. Our results demonstrated that phen (50 *μ*M) maintained PGSK fluorescence, similar to the effects of XN on Fe-SP treatment. These results showed that the XN possesses potent antioxidation and chelated intracellular iron in Fe-SP-induced H9c2 cardiomyoblasts ferroptosis.

### 3.3. Effects of XN on Ferroptosis-Induced Myocardial Cell Death

**Figure 3:**
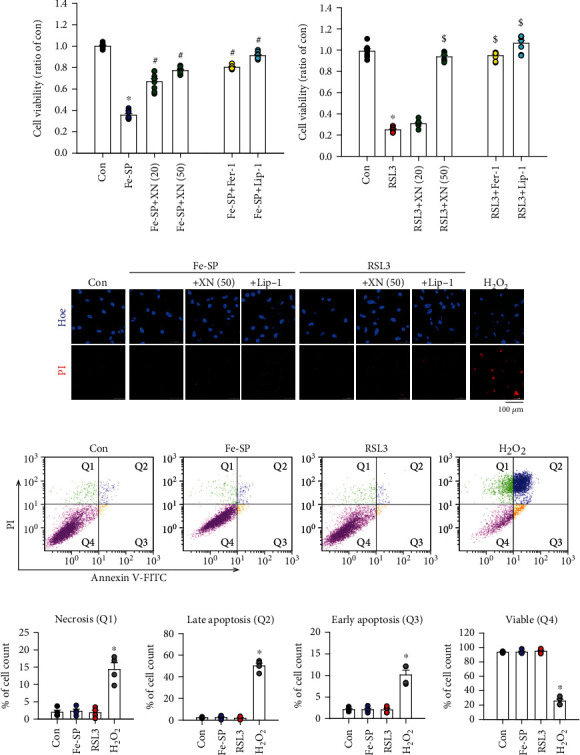
XN prevents ferroptosis-induced myocardial cell death. Treatment with (a) Fe-SP (2 *μ*M) or (b) RSL3 (0.5 *μ*M) for 24 h decreased the viability of neonatal cardiomyocytes examined using the MTT assay, and this effect was inhibited by cotreatment with XN (20 or 50 *μ*M) or a ferroptosis inhibitor (1 *μ*M of Fer-1, or 2 *μ*M of Lip-1) (*n* = 8). (c) Treatment of cardiomyocytes with Fe-SP (2 *μ*M) or RSL3 (0.5 *μ*M) decreased cell number, but did not induce chromatin condensation and necrotic cell death detected by confocal microscopy using Hoechst 33342/PI dye. XN (50 *μ*M) treatment abolished the Fe-SP- or RSL3-induced cell number reduction. H_2_O_2_ served as a positive control for cell death induction. Scale bars: 100 *μ*m. (d) Scatter plots of necrotic cells (Q1), late apoptotic cells (Q2), early apoptotic cells (Q3), and viable cells (Q4) distinguished by flow cytometric analysis using Annexin V-FITC and propidium iodide staining. (e) Percentage of necrotic cells, late apoptotic cells, and early apoptotic cells and viable were not significant difference between the Fe-SP- or RSL3-treated group and control group. H_2_O_2_ served as a positive control for cell death induction (*n* = 4). All data represent mean ± SEM. ^∗^*p* < 0.05, versus the control group; ^#^*p* < 0.05, versus the Fe-SP; ^$^*p* < 0.05, versus the RSL3. Con: control; Fer-1: ferrostatin-1; Lip-1: liproxstatin-1; Hoe: Hoechst 33342; PI: propidium iodide.

Neonatal cardiomyocytes have been considered as a very useful model for cardiac ventricular cells to study various cellular and molecular mechanisms. To verify our findings in the rat ventricular cell line H9c2, the cultured neonatal rat primary cardiomyocytes were subjected to Fe-SP and RSL3 to induce ferroptosis. Cardiomyocytes were treated with Fe-SP (2 *μ*M) for 24 h in the absence or presence of XN and then subjected for MTT assay to examine the cell viability. As shown in Figure 3, XN (20 or 50 *μ*M) exhibited the strongest protection against Fe-SP-induced ferroptotic cell death in cardiomyocytes (Figure 3(a), left panel). Cotreatment with RSL3 and XN (50 *μ*M) together also significantly reduced the ferroptotic cell death in cardiomyocytes (Figure 3(b), left panel). Furthermore, Fer-1 (1 *μ*M) and Lip-1 (2 *μ*M), a ferroptosis inhibitor, significantly reduced the Fe-SP- and RSL3-induced ferroptotic cell death, respectively (Figures 3(a) and 3(b), right panel). To confirm that the Fe-SP- and RSL3-induced cell death in the cardiomyocytes is not due to cell apoptosis and necrosis, the cell death was examined by confocal microscopy using Hoechst 33342 and propidium iodide (PI) and by flow cytometry using the Annexin V-FITC apoptosis kit, which includes Annexin V-FITC for apoptosis detection and PI for necrosis detection. Hoechst 33342/PI double staining showed that both Fe-SP and RSL3 reduced the number of cardiomyocytes, but did not induce chromatin condensation and necrotic cell death (Figure 3(c)). Treatment with XN can abolish the Fe-SP- and RSL3-reduced number of cardiomyocytes. H_2_O_2_ was used as a positive control for cell death induction.  Figure 3(d) shows the representative histograms of the Fe-SP- and RSL3-treated cardiomyocytes stained with Annexin V-FITC apoptosis kit. Treatment of cardiomyocytes with Fe-SP and RSL3 for 24 h did not significantly affect the number of necrotic cells, early and late apoptotic cells, and viable cells (Figure 3(e)). In contrast, treatment 1 h with 20 *μ*M H_2_O_2_, which served as a positive control for cell death induction, reduced the number of viable cells and increased the number of apoptotic and necrotic cells. These results indicate that Fe-SP- and RSL3-induced cardiomyocyte death was due to ferroptosis, but did not induce both apoptosis and necrosis.

### 3.4. Mechanism of XN on Fe-SP-Induced Ferroptosis in Cardiomyocytes

**Figure 4:**
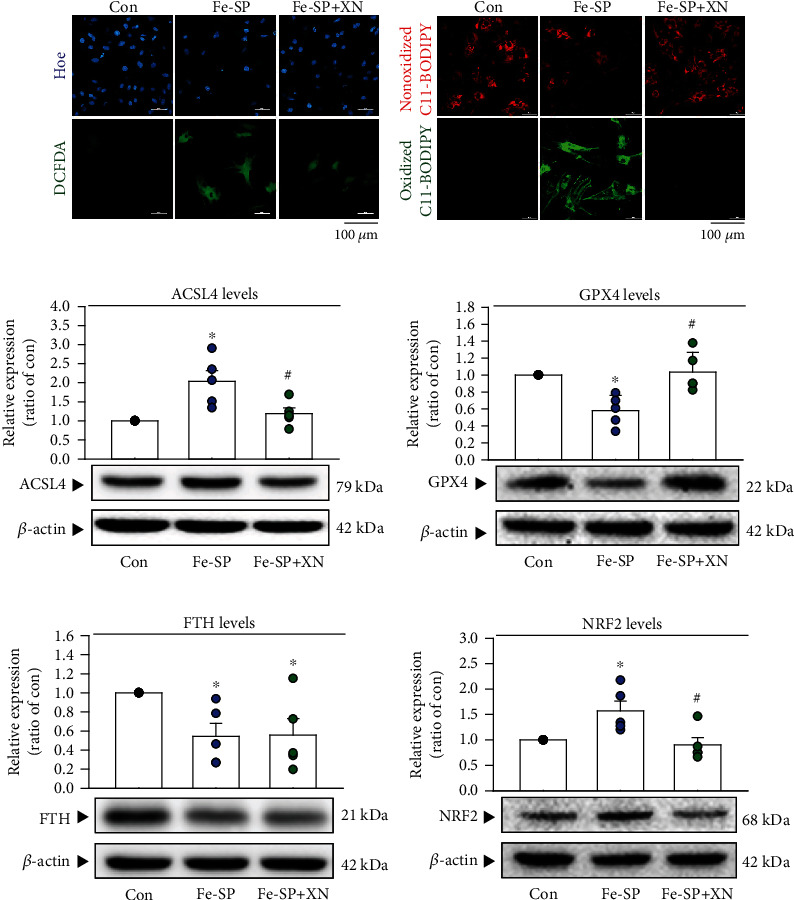
The mechanism of XN inhibited lipid peroxidation. (a) Representative fluorescence images of intracellular ROS using CM-H_2_DCFDA in neonatal cardiomyocytes. Treatment with Fe-SP (2 *μ*M) significantly increased the level of intracellular ROS (green, central panel), and this effect was abolished by XN (50 *μ*M) (right panel). Hoechst 33342 was used to identify the cell nuclei. Scale bars: 100 *μ*m. (b) Representative fluorescence images of lipid peroxidation using C11 BODIPY 581/591 in neonatal cardiomyocytes. Treatment with Fe-SP (2 *μ*M) significantly increased the level of lipid peroxidation (green, central panel), and this effect was abolished by XN (50 *μ*M) (right panel). Scale bars: 100 *μ*m. (c) Treatment with XN significantly inhibited Fe-SP-increased the level of ACSL4 protein (*n* = 5). (d) Treatment with XN increased the level of GPX4 protein as compared with the Fe-SP-treated group (*n* = 5). (e) Treatment with XN did not significantly affect the Fe-SP-reduced the level of FTH protein (*n* = 5). (f) Treatment with XN significantly inhibited the Fe-SP-increased level of NRF2 protein (*n* = 5). All data represent mean ± SEM. ^∗^*p* < 0.05, versus the control group; ^#^*p* < 0.05, versus the Fe-SP. Con: control; Hoe: Hoechst 33342.

Ferroptosis has been reported to the degree of unsaturation of polyunsaturated fatty acid in lipid bilayers, which are especially susceptible to peroxidation [32]. We examined whether XN could inhibit intracellular ROS production and decrease lipid ROS by confocal microscopy using the specific ROS dye CM-H_2_DCFDA and lipid ROS probe C11 BODIPY 581/591, respectively. As demonstrated in Figure 4, treatment with Fe-SP for 24 h significantly increased the intracellular ROS and lipid peroxidation level. Moreover, cotreatment with XN abolished intracellular ROS production (Figure 4(a)) and lipid peroxidation (Figure 4(b)) in cardiomyocytes. Recent studies showed that long-chain acyl-CoA synthetase 4 (ACSL4), which plays a key role in lipid biosynthesis and fatty acid degradation, is an essential enzyme for ferroptosis execution [33]. To further confirm the effect of XN on ferroptosis, we examine the protein level of ACSL4 detected by Western blot analyses. The results showed that the protein level of ACSL4 was significantly decreased in the XN-treated group as compared with the Fe-SP-treated group (Figure 4(c)). It has been reported that GPXs can use the glutathione system to prevent peroxides, of which GPX4 is the main neutralizer of lipid peroxides [34]. We further examined the protein levels of GPX4 by Western blot analyses. Treatment with Fe-SP significantly decreased the protein levels of GPX4, and this effect was prevented by cotreatment with XN (Figure 4(d)), whether XN also through changed iron-binding protein (ferritin heavy chain (FTH)) protect Fe-SP-induced ferroptosis. The results showed that treatment Fe-SP significantly decreased the protein level of FTH, and this reduction was not significantly affected by cotreatment with XN (Figure 4(e)). Nuclear factor-erythroid factor 2-related factor 2 (NRF2) is a stress-inducible transcription factor, such as RSL3 and iron can induce NRF2 expression in cancer cells and astrocytes, respectively [35, 36]. Whether the ferroptosis inhibition effects of XN were caused by inhibition of NRF2 was further evaluated. Treatment with Fe-SP for 2 h significantly increased the protein level of NRF2, and this effect was eliminated by cotreatment with XN (Figure 4(f)).

### 3.5. XN Protects the Heart against Ischemia-Reperfusion-Induced Ferroptosis

**Figure 5:**
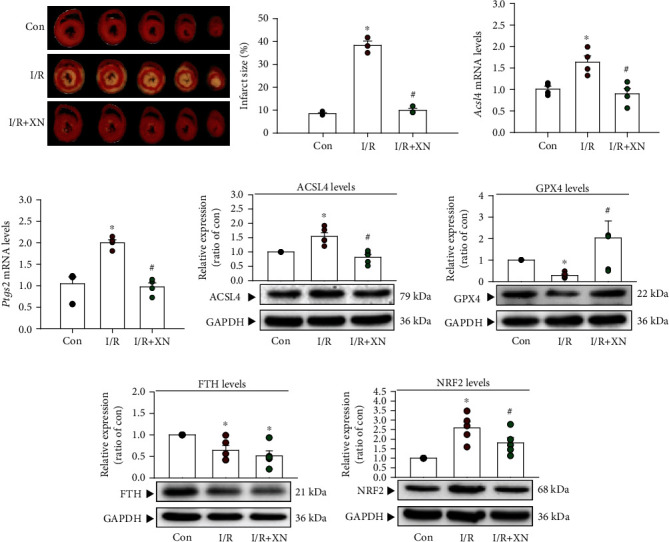
XN prevents the ischemia-reperfusion-induced ferroptosis in ex vivo hearts. (a) XN (5 *μ*M) significantly attenuated the I/R-induced increase of myocardial infarct size (*n* = 3). XN (10 *μ*M) significantly decreased the levels of *Ptgs2* (b) and *Acsl4* (c) mRNA in myocardial-treated with I/R, measured using quantitative PCR (*n* = 4). (d) Treatment with XN significantly inhibited the I/R-increased the level of ACSL4 protein enhancement (*n* = 5). (e) Treatment with XN increased the level of GPX4 protein as compared with the I/R-treated group (*n* = 5). (f) Treatment with XN did not significantly affect the I/R-reduced the level of FTH protein (*n* = 5). (g) Treatment with XN significantly inhibited the I/R-increased the level of NRF2 protein (*n* = 5). All data represent mean ± SEM. ^∗^*p* < 0.05, versus the control group; ^#^*p* < 0.05, versus the I/R. Con: control; I/R: ischemia-reperfusion.

Since in vitro study demonstrated that XN can protect cardiomyocytes against ferroptosis, we used Langendorff isolated heart preparations to investigate whether XN can reduce ferroptosis caused by I/R. To evaluate the effect of XN on I/R injury, the heart was subjected to 45 min of myocardial ischemia followed by 60 min reperfusion after TTC staining. The results showed that XN (5 *μ*M) significantly attenuated infarct size (white area) in ex vivo rat hearts (Figure 5(a)). In addition, XN (5 *μ*M) improved I/R-induced decrease of left ventricular developed pressure (LVDP) of isolated rat hearts (Supplementary Figure 1). Next, we applied the quantitative-PCR technique to examine the mRNA levels of *Acsl4* and *Ptgs2*, molecular markers of ferroptosis, in the *ex vivo* heart. The results showed that the mRNA levels of *Acsl4* and *Ptgs2* were significantly decreased in the heart cotreated with I/R and XN (10 *μ*M) together as compared with the I/R-treated alone (Figures 5(b) and 5(c)). The protein levels of ACSL4 also decreased in the heart cotreated with I/R and XN together (Figure 5(d)). GPX4 is a key enzyme, which protects cells from ferroptosis by removing lipid hydroperoxide [37]. We also observed that the protein levels of GPX4 were significantly increased in hearts cotreated with I/R and XN together as compared with the heart treated with I/R alone (Figure 5(e)). In addition, I/R significantly decreased the protein level of FTH. However, cotreatment with XN and I/R together did not significantly affect the protein levels of FTH as compared with treatment with I/R alone (Figure 5(f)). Similar to the results from the study conducted in cardiomyocytes, treatment with I/R and XN together significantly decreased the NRF2 protein level as compared with treatment with I/R alone (Figure 5(g)). Taken together, these results suggested that XN could regulate the protein levels of GPX4, thereby protecting against the I/R-induced ferroptotic cell death in the ex vivo heart.

## 4. Discussion

In the present study, we identified XN as a potent natural compound that protects cardiomyocytes from ferroptosis inducers- and I/R-induced ferroptotic cell death. XN protects H9c2 cardiomyoblasts from oxidative stress (lipid peroxidation and ROS) when they are exposed to RSL3- and Fe-SP-induced ferroptosis. XN is a powerful inhibitor of iron accumulation in Fe-SP-induced H9c2 cardiomyoblasts. Mechanistically, XN prevents the protein levels of GPX4 reduction and decreases the protein levels of NRF2 in the Fe-SP-treated cardiomyocytes. Furthermore, XN reduced the heart infarct size up to 95% when the rat hearts were exposed to I/R, likely due to reduction of the mRNA levels of *Ptgs2* and *Acsl4* as well as the protein levels of ACSL4 and NRF2 and modulation of the protein levels of GPX4 to attenuate cardiac I/R-induced ferroptosis. Additionally, a large number of compelling pieces of experimental evidence indicated the protective role of XN for cardiovascular diseases. For example, XN plays a potential protective effect in the aged cardiovascular system [38]. Another study suggests that XN attenuated isoprenaline-induced cardiac hypertrophy and fibrosis [39]. In the past few years, XN has been found to protect against IRI in the liver and brain, suggesting that this natural substance can be as a potential candidate for preventing the IRI in the heart [40, 41].

RSL3, a ferroptosis inducer, directly acts on GPX4 and inhibits its activity. Although RSL3 can increase the levels of ROS, lipid peroxidation, and *Ptgs2* mRNA to induce ferroptosis in H9c2 cardiomyoblasts, Fe-SP can induce intracellular iron overload, but not RSL3 in our study. Intracellular iron accumulation is considered a contribution to the execution of ferroptosis during cardiac I/R. In the present study, using neonatal cardiomyocytes treated with Fe-SP to mimic the pathophysiology of myocardial I/R injury in vitro, we evaluated the contribution of XN to prevent ferroptosis. In addition, Park et al. showed that undifferentiated C2C12 and H9c2 cells were highly sensitive to RSL3-induced ferroptosis, while differentiated C2C12 cells and neonatal rat cardiomyocytes were relatively resistant to ferroptosis. This is because myocyte differentiation induces some changes in cell signaling pathways to acquire resistance to RSL3-induced ferroptosis [10].

GPX4, a key antioxidant enzyme in the regulation of ferroptosis, can directly detoxify lipid hydroperoxides generated by ROS. In a previous study, it has been reported that downregulation of GPX4 during the early and middle stages of myocardial infarction contributes to ferroptosis in cardiomyocytes upon metabolic stress such as cysteine deprivation. Depletion or inhibition of GPX4 using specific siRNA or RSL3, respectively, resulted in accumulation of lipid peroxide, leading to ferroptosis in H9c2 cardiomyoblasts [10]. Besides, ectonucleotide pyrophosphatase/phosphodiesterase 2, a lipid kinase involved in lipid metabolism, overexpression protects H9c2 cardiomyoblasts from erastin-induced ferroptosis through modulating GPX4, ACSL4, and NRF2 expression [42]. Evidence has shown that NRF2 regulates ferroptosis primarily by directly affecting the GPX4 synthesis and function [43]. It is consistent with our findings that Fe-SP-induced cardiomyocytes and I/R-induced hearts lead to an increase in the protein levels of NRF2 and a decrease in the protein levels of GPX4, and these effects can be abolished by XN, although XN has been demonstrated to be active of antioxidants through the NRF2/heme oxygenase-1 signaling pathway. However, a study found that doxorubicin-induced cardiomyopathy results in the accumulation of nonheme iron via heme degradation by NRF2-mediated upregulation of heme oxygenase-1 [14]. Recent studies have indicated that heme oxygenase-1 plays a positive association role in ferroptosis by iron accumulation [14, 44].

Some flavonoids efficiently chelate trace metals like copper and iron which are potential enhancers of ROS [45]. In the present study, we found that one of the mechanisms of action of XN in preventing ferroptosis is iron chelation. Consistent with our results, a study shows that XN can reduce iron accumulation in opisthorchis viverrini-induced cholangiocarcinogenesis [46]. In addition, Gao et al. found that inhibition of ferroptosis by deferoxamine, an iron chelator, can reduce the release of lactate dehydrogenase, improve cardiac function, and protect heart tissue from IRI [47]. Another iron chelator, dexrazoxane, can protect the heart against IRI-induced ferroptosis [14]. In the present study, we showed that XN can reduce iron accumulation and inhibit ROS production in the Fe-SP-treated H9c2 cardiomyoblasts.

Lipid peroxidation is the key downstream feature of ferroptosis. Various lipids, such as phosphatidylcholine and phosphatidylethanolamine, are responsible for ferroptosis-induced lipid peroxidation. Peroxidized phosphatidylethanolamine species have been identified as predictive biomarkers for ferroptosis. Recent studies have shown that using gas cluster ion beam secondary ion mass spectrometry imaging can map PEox at ferroptotic H9c2 cardiomyoblasts [48]. In addition, LC-MS/MS analysis revealed a dramatic increase in PEox species in mitochondria in response to RSL3 and cardiac I/R [9]. A previous study showed that XN markedly decreases the level of lipid peroxidation and ROS production in rat liver [49]. Consistent with our results shown that XN inhibited RSL3- and Fe-SP-induced lipid peroxidation, MDA, and ROS enhancements in H9c2 cardiomyoblasts as well as Fe-SP-induced lipid peroxidation in cardiomyocytes. In addition, ACSL4 acts as an essential component for ferroptosis execution and dictates ferroptosis sensitivity by shaping cellular lipid composition. In the present study, we found that XN inhibited Fe-SP- and I/R-induced upregulation of ACSL4 in cardiomyocytes and ex vivo hearts. A study has shown that XN possesses glutathione levels and glutathione peroxidase activities to reduce lipid peroxidation on carbon tetrachloride-induced acute liver injury in rats [50]. Another report has shown that XN markedly reduced 4-hydroxynonenal and increased glutathione levels in the hepatic I/R-injury in mice [40]. In the present study, we found that XN can modulate the protein levels of GPX4 from the Fe-SP-induced cardiomyocytes and the I/R-induced hearts. Altered expression of GPX4 may play an important role in the protection of cardiomyocytes against ferroptosis.

Ferroptosis induced by I/R or ferroptosis inducer may show some biochemical characteristics as well as changes in protein and gene levels. The lipid peroxidation requires ACSL4-mediated conversion of arachidonic acid (AA) to AA-CoA. Importantly, the upregulation of ACSL4 levels is a biomarker of ferroptosis sensitivity [6]. Some studies have indicated that degradation of GPX4 protein can also be observed in ferroptosis-sensitive cells in response to erastin and RSL3 [51, 52]. In addition, the upregulation of *Ptgs2* mRNA is used as a biomarker of ferroptosis in mice exposed to erastin or RSL3. However, the upregulation of PTGS2 is also observed under nonferroptotic conditions, such as inflammation [53]. Moreover, some biochemical hallmarks of ferroptosis are used to distinguish ferroptosis and other types of cell death, such as accumulation of cellular iron or induction of lipid peroxidation. Unfortunately, the biomarkers of ferroptosis are also present in other types of cell death or pathological conditions. Therefore, more specific biomarkers are needed to detect ferroptotic cell death in the future [54].

## 5. Conclusions

Our results suggest that XN could chelate iron and inhibit ROS and lipid peroxidation in the Fe-SP- or RSL3-induced ferroptotic cell death in cardiomyocytes. Moreover, our ex vivo data support that XN can prevent ferroptosis during cardiac I/R by reducing the expression of *Acsl4* and *Ptgs2* mRNA, reducing the expression of ACSL4 and NRF2 protein, and modulating the expression of GPX4 protein.

## Data Availability

All materials are available by the corresponding author.

## Supplementary Materials

Supplementary Materials Supplementary Figure 1: xanthohumol (XN) tended to suppress the I/R-induced decrease of left ventricular developed pressure (LVDP) of isolated rat heart. A latex balloon filled with Milli-Q water was inserted into the LV to record LV pressure. LV pressure was recorded by Power Lab system and Chart 8 software (ADInstruments, New South Wales, Australia). LVDP was calculated as the difference between maximal systolic and end-diastolic pressures. The hearts were exposed to 45 min no-flow ischemia of global and 60 min of reperfusion, showing improved postischemic LV pressure recovery upon treatment with XN (5 *μ*M) at 40 min. Supplementary Figure 2: the entire gel pictures of Figure 4 in the text. (a) Corresponding to Figure 4(c). (b) Corresponding to Figure 4(d). (c) Corresponding to Figure 4(e). (d) Corresponding to Figure 4(f). The protein marker and the molecular weight of each protein were indicated. Supplementary Figure 3: the entire gel pictures of Figure 5 in the text. (a) Corresponding to Figure 5(d). (b) Corresponding to Figure 5(e). (c) Corresponding to Figure 5(f). (d) Corresponding to Figure 5(g). The protein marker and the molecular weight of each protein were indicated.
