# Supplementary material for: Xanthohumol Protects the Rat Myocardium against Ischemia/Reperfusion Injury-Induced Ferroptosis
Source: Oxid Med Cell Longev. 2022 Jan 17;2022:9523491. doi: 10.1155/2022/9523491 (PMC8786462; doi:10.1155/2022/9523491)
Supplement: Supplementary Materials — Supplementary Figure 1: xanthohumol (XN) tended to suppress the I/R-induced decrease of left ventricular developed pressure (LVDP) of isolated rat heart. A latex balloon filled with Milli-Q water was inserted into the LV to record LV pressure. LV pressure was recorded by Power Lab system and Chart 8 software (ADInstruments, New South Wales, Australia). LVDP was calculated as the difference between maximal systolic and end-diastolic pressures. The hearts were exposed to 45 min no-flow ischemia of global and 60 min of reperfusion, showing improved postischemic LV pressure recovery upon treatment with XN (5 μM) at 40 min. Supplementary Figure 2: the entire gel pictures of Figure 4 in the text. (a) Corresponding to Figure 4(c). (b) Corresponding to Figure 4(d). (c) Corresponding to Figure 4(e). (d) Corresponding to Figure 4(f). The protein marker and the molecular weight of each protein were indicated. Supplementary Figure 3: the entire gel pictures of Figure 5 in the text. (a) Corresponding to Figure 5(d). (b) Corresponding to Figure 5(e). (c) Corresponding to Figure 5(f). (d) Corresponding to Figure 5(g). The protein marker and the molecular weight of each protein were indicated. [file 9523491.f1.docx]

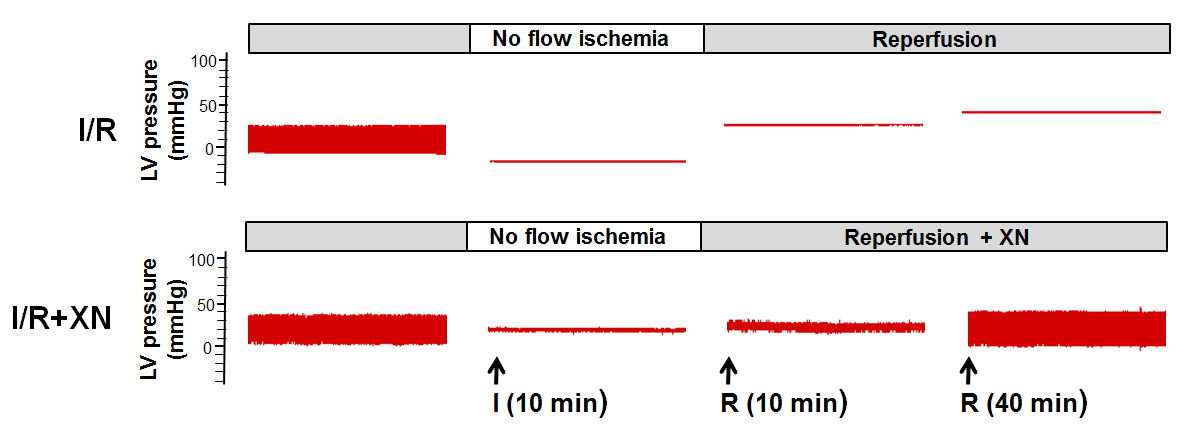


**Supplementary Figure 1:** Xanthohumol (XN) tended to suppress the I/R-induced decrease of left ventricular developed pressure (LVDP) of isolated rat heart. A latex balloon filled with Milli-Q water was inserted into the LV to record LV pressure. LV pressure was recorded by Power Lab system and Chart 8 software (ADInstruments, New South Wales, Australia). LVDP was calculated as the difference between maximal systolic and end‐diastolic pressures. The hearts were exposed to 45 min no-flow ischemia of global and 60 min of reperfusion, showing improved post-ischemic LV pressure recovery upon treatment with XN (5 μM) at 40 min.


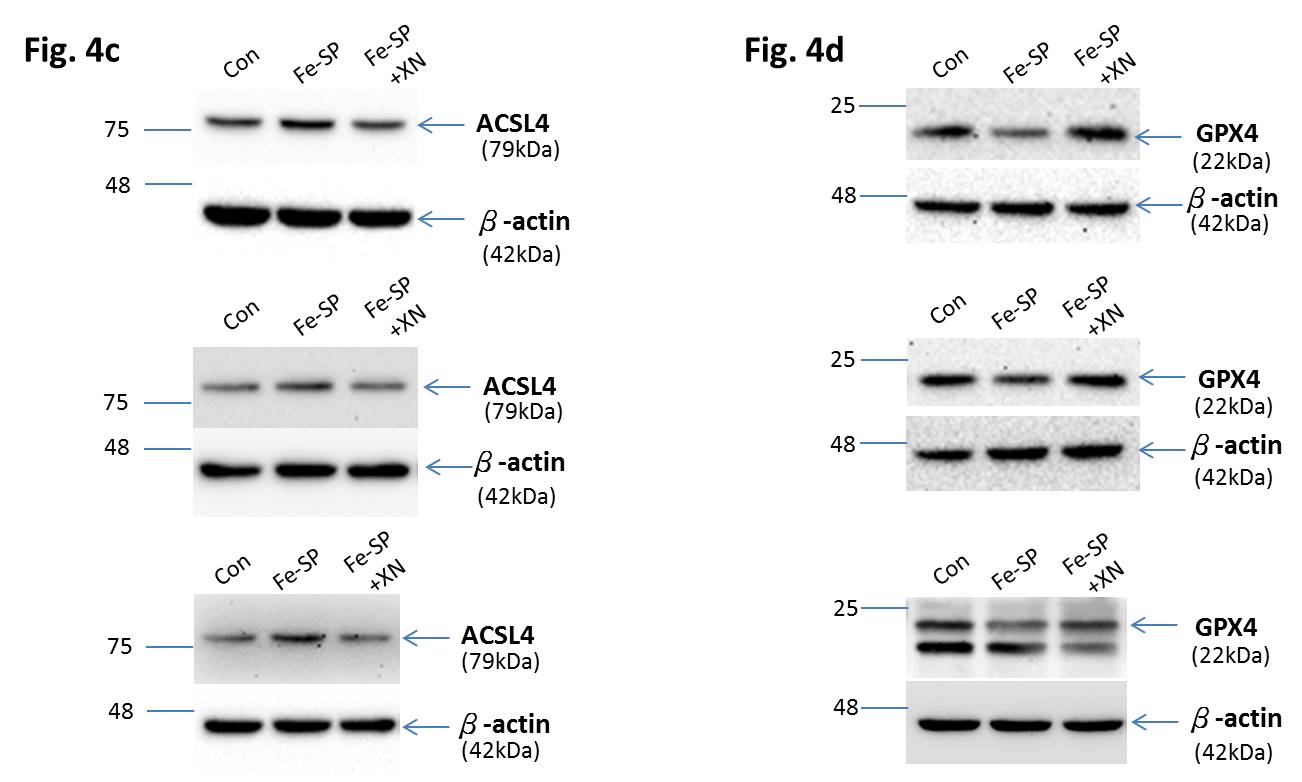


(B)

(A)

(D)

(C)


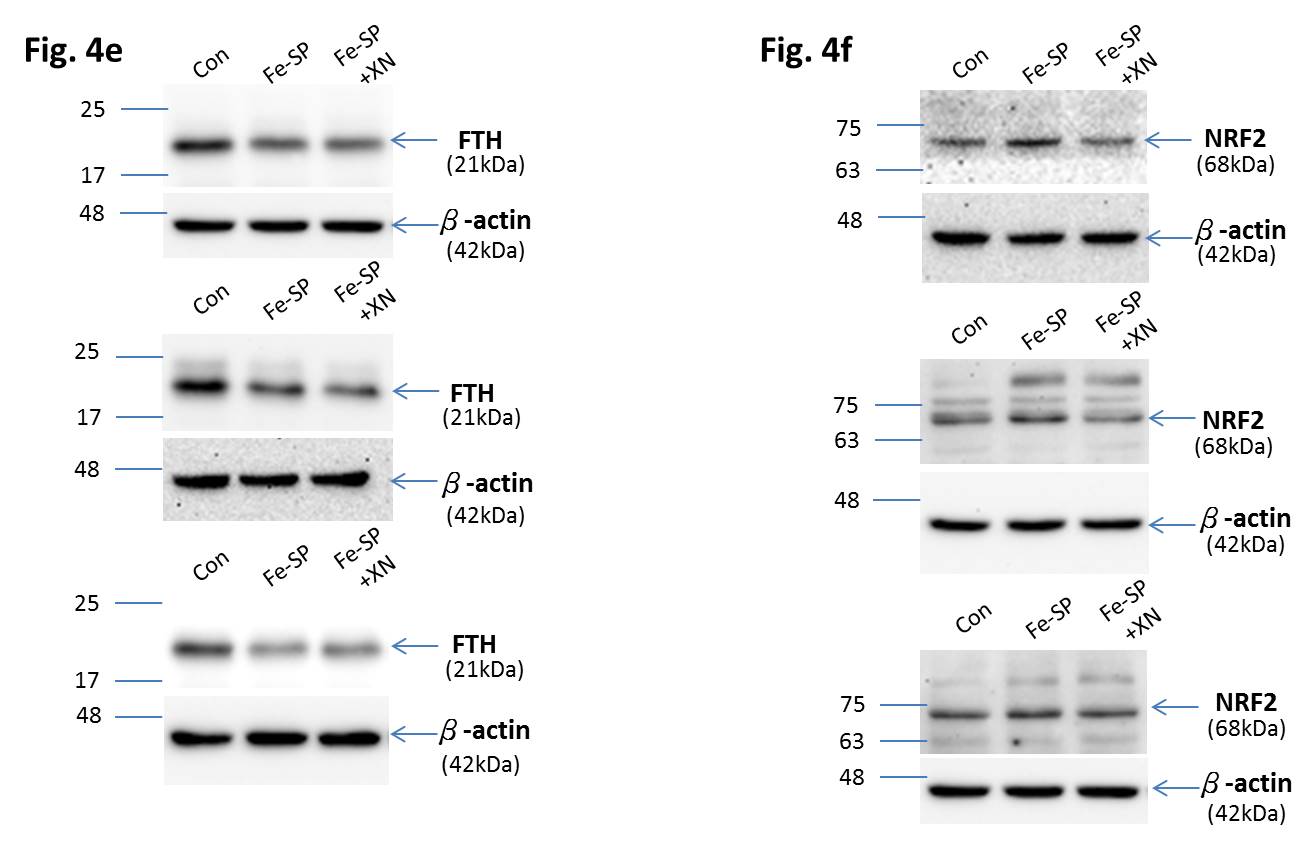


**Supplementary Figure 2:** The entire gel pictures of Figure 4 in the text. (A) Corresponding to Figure 4c. (B) Corresponding to Figure 4d. (C) Corresponding to Figure 4e. (D) Corresponding to Figure 4f. The protein marker and the molecular weight of each protein were indicated.


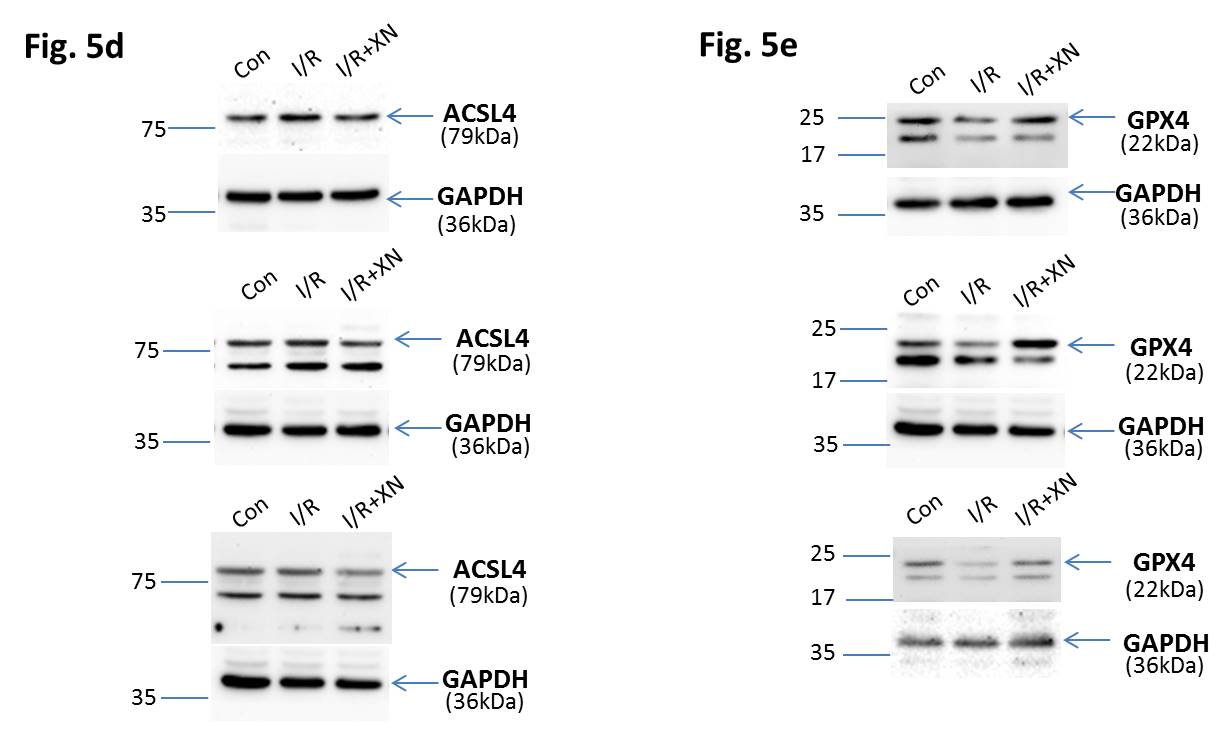


(A)

(B)


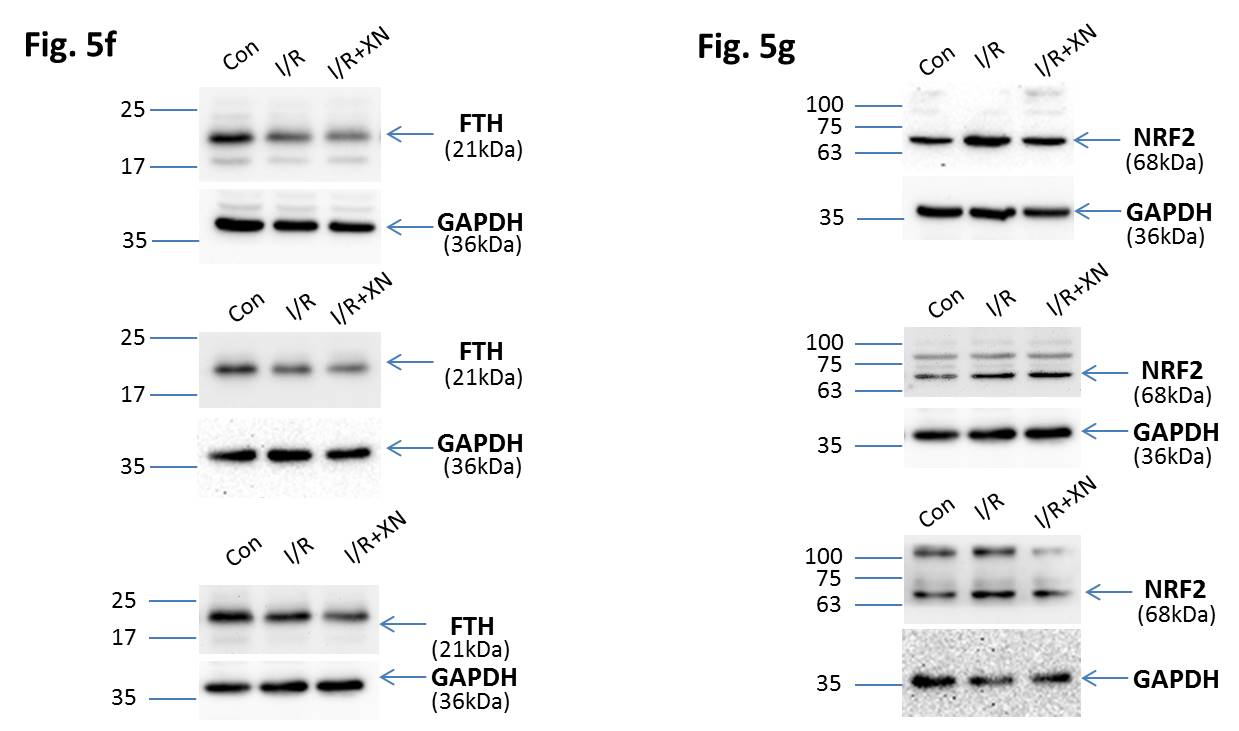


(C)

(D)

**Supplementary Figure 3:** The entire gel pictures of Figure 5 in the text. (A) Corresponding to Figure 5d. (B) Corresponding to Figure 5e. (C) Corresponding to Figure 5f. (D) Corresponding to Figure 5g. The protein marker and the molecular weight of each protein were indicated.
